# Cross-cultural acceptability and utility of the strengths and difficulties questionnaire: views of families

**DOI:** 10.1186/s12888-016-1063-7

**Published:** 2016-10-12

**Authors:** Paula Kersten, Margaret Dudley, Shoba Nayar, Hinemoa Elder, Heather Robertson, Robyn Tauroa, Kathryn M. McPherson

**Affiliations:** 1 School of Health Sciences, University of Brighton, Brighton, UK; 2 Faculty of Health & Environmental Sciences, AUT University, Auckland, New Zealand; 3 School of Psychology, University of Auckland, Auckland, New Zealand; 4 Centre for Person Centred Research, AUT University, Private Bag 92006, Auckland, 1142 New Zealand; 5 Te Whare Mātai Aronui, Te Whare Wānanga o Awanuiārangi, Auckland, New Zealand; 6 Chief Executive Health Research Council of New Zealand, 110 Stanley St, Auckland, New Zealand

**Keywords:** Strengths and Difficulties Questionnaire, SDQ, Culture, Validity, Parents

## Abstract

**Background:**

Screening children for behavioural difficulties requires the use of a tool that is culturally valid. We explored the cross-cultural acceptability and utility of the Strengths and Difficulties Questionnaire for pre-school children (aged 3–5) as perceived by families in New Zealand.

**Methods:**

A qualitative interpretive descriptive study (focus groups and interviews) in which 65 participants from five key ethnic groups (New Zealand European, Māori, Pacific, Asian and other immigrant parents) took part. Thematic analysis using an inductive approach, in which the themes identified are strongly linked to the data, was employed.

**Results:**

Many parents reported they were unclear about the purpose of the tool, affecting its perceived value. Participants reported not understanding the context in which they should consider the questions and had difficulty understanding some questions and response options. Māori parents generally did not support the questionnaire based approach, preferring face to face interaction. Parents from Māori, Pacific Island, Asian, and new immigrant groups reported the tool lacked explicit consideration of children in their cultural context. Parents discussed the importance of timing and multiple perspectives when interpreting scores from the tool.

**Conclusions:**

In summary, this study posed a number of challenges to the use of the Strengths and Difficulties Questionnaire in New Zealand. Further work is required to develop a tool that is culturally appropriate with good content validity.

## Background

Children as young as three are now being assessed for behavioural and emotional problems in many countries. This universal approach to screening is underpinned by the knowledge that such problems can impact upon children’s transition into primary school [1, 2], affect their educational achievement, [3] and lead to problems in middle-childhood and adulthood [4, 5], including depression and anti-social behaviours [6, 7]. The most commonly used tool for screening is the Strengths and Difficulties Questionnaire (SDQ), completed by parents and/or teachers [8, 9]. Our recent systematic review showed the evidence for a number of psychometric properties is strong [10], however, evidence of cultural validity is limited. One study examined measurement invariance with respect to ethnicity between British Indian and British white children using data from the 1999 and 2004 British Child and Adolescent Mental Health Surveys (A. Goodman, Patel, & Leon, 2010). All parents completed the English version of the SDQ and the multi-group confirmatory factor analyses provided evidence of acceptable fit to the parent and teacher SDQ across ethnicity. Only one study was identified that specifically addressed the tool’s content and cultural validity with parents [11]. This study was carried out in Aboriginal community-controlled health services. Participants in this study reported that the use of a questionnaire as opposed to a general conversation or interview was culturally inappropriate and problematic for those with literacy issues. Inter-relationships with peers were considered of less importance than relationships with family and participants felt that many important aspects of children’s behaviour and emotions were not covered by the SDQ. They also reported that the SDQ might not be completed honestly for fear of use of the data by other services or answers reflecting badly on their parenting skills.

Enquiries through the SDQ website *Youth in Mind* have revealed that SDQ translations are carried out in consultation with the SDQ team, and that this includes forward and backward translations (personal communication, www.youthinmind.info). This may be one reason why we only identified six articles that reported on the language validation of the SDQ into Arabic, Maltese, Bangla, Urdu and Chinese [12–16] in our review [10]. Some of these studies reported that parents had difficulties with literacy and required support to complete the questionnaire [14, 16] and that some questions required cross-cultural adaptations to make them more acceptable [16]. Furthermore, one paper highlighted problems with the provided specific examples that outlined the Chinese version of the SDQ [17].

These are important issues in the context of New Zealand, which is a country with a sizeable indigenous (Māori) and immigrant population [18]. In the most recent census 14.9 % of the New Zealand population identified as Māori, with almost half of these (46.5 %), also identifying with another ethnic group [18]. Indeed, 22.8 % of all New Zealand children identify with more than one ethnic group. The census also showed that 25.2 % of the population were born overseas, a third of these in Asia. This percentage is even greater in Auckland where 39.1 % of the population were born overseas. By comparison, in England (where the SDQ was first developed) 14 % of residents are born overseas [19]. These statistics show that New Zealand, and Auckland in particular is a multi-cultural society, impacting upon values, ways of living and languages spoken. It is therefore paramount that measurement tools used can be utilised across diverse groups. This study aimed to examine the cross-cultural acceptability and utility of the SDQ with parents/whānau (extended family groups including parents) of pre-school children (aged 3 to 5).

## Methods

Focus groups were utilised as a prompt for identification of key concerns and to promote shared experiences for stimulating deeper thinking and/or debate on a topic [20, 21]. Those participants who preferred an interview or for other reasons were unable to partake in a focus group were offered individual interviews.

Parents were recruited via early mainstream and Māori childhood education centres, the HIPPY Foundation (http://www.greatpotentials.org.nz/), our research centre register of people who had expressed an interest to hear about ongoing projects, Plunket (New Zealand’s largest provider of support services for the development, health and wellbeing of children under 5), snowball techniques, and Facebook. All potential participants received an information sheet about the study (provided in different languages e.g. English, Te Reo Māori and Tongan), had opportunities to discuss the study with the researchers, and if willing to take part were asked to provide written informed consent. All participants were asked to keep confidential the topics discussed as well as names of people who took part. In addition, they were assured the research team would protect their confidentiality through anonymising of transcripts, the use of ID numbers rather than names in reporting, and when discussing the work with others (e.g. stakeholders).

Purposeful sampling [22] through the aforementioned routes was used to recruit participants who were diverse in terms of demographic profile (e.g. ethnicity, number of children, age and gender), location (urban and rural areas), and early childhood education utilisation (e.g. not attending; attending mainstream and Māori/Pacific centres). Sample size was informed by methodological literature, suggesting focus group sizes of 4–8 participants are most appropriate [20] and that the majority of codes can be identified after analysis of 12 transcripts [23]. In addition, sample size was informed by our desire to achieve a diverse sample.

Focus groups and interviews were conducted at a place identified as convenient and culturally appropriate by participants, and lasted approximately 60 to 90 min. Each focus group had a group facilitator and co-facilitator (who also acted as an observer and note-taker). Each focus group and interview was facilitated by the most appropriate researcher, aiming for ethnic match wherever possible; including Māori, Asian, Pacific, New Zealand European and recent immigrant researchers. Translators were used for interviews and focus groups that were carried out in languages other than English (Chinese, Korean, Kirabati and Tongan). Refreshments were available prior to the commencement of each group, allowing an opportunity for a brief period of informal social interaction between participants on arrival [20]. In addition, we included karakia (prayer/blessing), mihimihi (brief introductions to establish relationships) and the whakatau process (formal welcome, i.e. allowing hosts to open the meeting) in the Māori focus groups and interviews. These are well established cultural protocols and rituals of encounter that are critical in safely engaging and involving Māori whānau [24]. The facilitators briefly explained their roles and offered participants the opportunity to clarify any last minute points about the research purpose or group procedure.

The focus groups and interviews followed a similar format to that previously used with Aboriginal Communities in Australia [11] (Table 1), commencing with exploration of participants’ views about children’s behaviour and emotional development, both generally and in relation to their own children. Prompts were then used to explore how strengths and difficulties would typically be expressed, without reference to the SDQ. This was done to facilitate an open and unbiased discussion. Subsequently, people were provided with the English version of the SDQ (for children aged 3 and 4). This is the version parents would be asked to complete as part of the Before School Check and therefore the version that should be considered. Content validity was examined through discussion of the relevance of questions and topics in the SDQ as well as those people felt were missing. Cultural equivalence [25] of the SDQ was examined by exploring the meaning different ethnic groups attached to the construct, sentences, words and response options. Similarly, participants were asked how responses from different raters should be interpreted (e.g. a mother and father, or a parent and teacher) and whether there were issues in terms of linguistic equivalence.

**Table 1:** Focus group and interview format

a) Participants’ views were explored concerning children’s behaviour and emotional development and how strengths and difficulties would typically be expressed.
b) Participants were then given an overview of the purpose and scoring of the SDQ and asked to read the questionnaire.
c) Participants were asked to discuss each SDQ item and consider whether
a. it taps into important indicators of behaviour and emotional development in pre-school children;
b. the meaning of the question and response category is clear;
c. the language used in the question and response categories is understood and appropriate;
d. whether parents with English as a second language need greater support and whether there is a need for translation;
e. if more than one teacher is involved with the child’s education who should complete the SDQ-Teacher version; and
f. how to interpret multi-informant results (e.g. parent and teacher/more than one teacher/two parents), especially if they differ in the assessment of the child.
d) Participants were also asked if there are important aspects of children’s behaviour and emotional development that are not covered by the questionnaire and which they considered critical.
e) Participants were asked if they would complete or had completed the SDQ as part of a Before School Check (why and why not), and whether they would have/had any support needs to complete it.

Focus groups and interviews were audio-taped and transcribed verbatim and IDs were allocated to each participant. Thematic analysis using an inductive approach was employed, following the methods set out by Braun and Clark [26]. This process began with familiarisation with the data by listening to recordings, reading and re-reading transcripts, and noting down and discussing initial ideas. Initial codes were then assigned to sections of text in each transcript. Codes for the different key ethnic groups (New Zealand European, Māori, Pasifika, Asian and other immigrants) were then compared, before grouping them into themes of importance (guided by cultural expertise within the research team). Themes were named and defined in a codebook, reviewed by the research team and refined when necessary. Rigour was ensured by regular meetings between the data analysts to discuss interpretation of data, and seeking feedback from the wider project management team [27]. We checked data saturation was achieved during the analysis and defined this as the point in data collection and analysis when new information produced little or no change to the codebook [23]. The interpretation of the analysis was not checked with study participants as member checking has been found to be challenging, for example when participants change their views over time or feel exposed by the analysis [28]. Drafting the study report and analysis were carried out iteratively. Illustrative quotes are provided in the results section (IDs are shown as IDx_y where x denotes the number of the focus group and y the ID number of the participant, or as IDz which denotes a participant with ID number z who took part in an individual interview).

## Results

In total 65 parents/whānau took part in the study. Seven ethnic-specific focus groups with 31 parents/whānau and two mixed-ethnicity groups with 16 parents were conducted, with an additional 18 individual interviews undertaken. The majority of parents/whānau were female (85 %), mothers (80 %), fathers (15 %), and aged between 26 and 45 (83 %). There was a widespread ethnic mix of parents/whānau and their children, which was a specific aim of the recruitment procedure (Table 2). Many of the parents (18 %) reported more than one ethnicity for their children and the vast majority of children had siblings (78 %; mean [SD] range 2.2 [1.6] 0 to 6). Many parents (51) reported their child attended some form of pre-school (78 %), of which 14 % attended Te Kohanga Reo or Puna (Māori pre-schools). Of the 11 parents (17 %) who stated their child did not attend, five were supported at home in providing pre-school education by a HIPPY Foundation facilitator. We achieved a good spread of participants from more rural areas west and North of Auckland (25 %), South, East and Central Auckland (19 %, 10 % and 32 % respectively), and from the North Shore of Auckland (14 %). Thus, participants came from both affluent and less affluent areas. Five key themes were identified (Table 3), which were prevalent amongst all ethnic groups. Therefore, rather than presenting the numbers of people from the different groups that specifically reported issues raised (frequentist reporting of qualitative data being considered by many to be inappropriate [26]), we indicate the ethnic group the participant identified with for each quote.

**Table 2:** Parent/whānau and their pre-school child ethnicity (*n =* 65)

Ethnicity (main)^a^	Frequencies (%)
- Māori	13 (20 %)
- NZ European	18 (27.7 %)
- Pacific Island	13 (20 %)
- Asian	18 (27.7 %)
- European	2 (3.1 %)
- Other	1 (1.5 %)
Child’s ethnicity (main)^b^	
- Māori	15 (23.1 %)
- NZ European	15 (23.1 %)
- Pacific Island	14(21.5 %)
- Asian	18 (27.7 %)
- European	1 (1.5 %)
- Other	1 (1.5 %)
- Missing	1 (1.5 %)
Child’s ethnicity (2nd reported)	
- NZ European	7 (10.8 %)
- Pacific Island	2 (3.1 %)
- Asian	3 (4.6 %)
Child’s ethnicity (3rd reported)	
- NZ European	3 (4.6 %)
- Pacific Island	1 (1.5 %)
Child’s ethnicity (4th reported)	
- Asian	1 (1.5 %)

^a^Three parents reported mixed ethnicity (Māori & NZE, Māori & Pacific Island, Māori and Asian)

^b^1 parent reported 4 ethnicities for the child, 3 parents reported 3 ethnicities, 8 parents reported 2 ethnicities

**Table 3:** Qualitative study themes

Confusion versus Clarity
There were mixed views about the perceived purpose and usefulness of the SDQ
Context Matters
Seeing data from the questionnaire in the wider context in which the child and family/whānau live, including cultural values/practices and context of history and colonisation
Questions and More Questions
Completing the questionnaire is a challenging process and generated a number of questions when reading through and reflecting on the questionnaire
Timing of the questionnaire
Children at the age of 4 and 5 constantly grow and change
Perspectives
Different people might provide a different perspective of the child

### Confusion versus clarity

This theme encapsulates the purpose and usefulness of the tool, as perceived by those completing it and what would be done with the data. While a number of people suggested the test was well intended, most parents were unclear about the purpose of the SDQ and raised concerns about the adequacy of services to which children would be referred. This lack of understanding led to parents not providing truthful answers, fearing telling the truth would reflect badly on them.

*“I wasn’t quite happy about the survey that we did, ‘cause I didn’t know that she was trying to figure out if there was anything wrong with my daughter”.* (Māori participant ID2_7)

*“My concern about when I was filling it out is really more to do with what’s going to happen to me ticking these boxes. I mean what happens if I do tick a box that says often loses temper, certainly true, and can be spiteful to others, certainly true, I mean what happens to that information, what’s the consequence of that happening, and would that change the box that I end up ticking because of my parental fear about, I don’t know”.* (NZE participant ID11_3)

For some participants, the SDQ questions assisted them to recognise their child’s behaviours and emotions and helped them to develop strategies to build on their strengths. Others, however, did not feel it was useful to identify their child’s behavioural and emotional difficulties, although it provided an opportunity to reflect on their behaviour.

*“That’s one positive thing I think I got from this questionnaire is it made me think and explore my child’s behaviour and his thoughts a bit more”* (Indian participant ID1_4)

### Context matters

Participants discussed the importance of the wider context in which the child and family/whānau live when answering these questions. Overall, participants felt that the questionnaire did not allow room for them to explain their concerns or provide a context for why they had given their respective scores. For example, parents highlighted that their children’s behaviour and emotional expressions could vary across settings and that the questionnaire did not allow them to state when the behaviour was considered normal or not.

*“It’s context as well, like this one that says often unhappy, depressed or tearful, well like at home at the moment that’s like* < child’s name > *all the time. She’s always upset about everything, but when she’s, I know that when she’s at preschool she’s perfectly happy and normal”.* (NZE participant ID11_3)

*“When it comes to your second child you’re like, ‘no I don’t have any problems’ unless it’s a serious problem because you’ve been through it before, it’s really nothing… and then you get better at recognising what the serious issues are as opposed to the tiny little issues that don’t really matter”.* (Translated: Chinese participant ID10_2)

Cultural values and practices were reported to be important contextual factors when completing the questionnaire by parents from all ethnic groups apart from New Zealand European participants. Some commented that what could be perceived as a negative answer, could in fact be explained through a cultural lens that made the response acceptable. Consequently, considering the child in their cultural context was important and the tool did not allow for this to be explored or recorded. For example:

*“One of the questions here, gets along better with adults than with other children. Looking from a cultural perspective… Indian children are taught to be part of the family all the time. So if we go into a new situation, new place, they will always hang with the adults first and then they go with the children, with the group*”. (Fijian participant ID1_8)

Indeed, some participants reported that cultural values and perspectives might impact upon people’s willingness or ability to be open when answering the questions, and that this may lead to tension:

*“There are times that it may seem hard when I am shy to say that my child was born with that specific difficulties. I feel that if I say so it will be a big rumour to my family. Therefore I will never talk about it. That will prevent me from completing the form according to what is needed”. (*Translated: Tongan participant ID3_3)

In addition, the context of colonisation and history was discussed by Māori participants. Some participants believed children’s behaviours were a direct consequence of colonisation and therefore, it was important to view their children within an historical context.

*“Māori are more understanding of, oh yeah, you know, we’ve got a better instinct when it comes to speaking with each other. We know what we’ve been through, we know what our people have been through, we know that we come with more than just what you’re seeing at the door, you’ve got your tūpuna <* ancestors *> and everything that comes with you and your baby”.* (Māori participant ID5_3)

Also evident was the impact of discrimination and the use of a deficit model that judges Māori tamariki (children) and their whānau. As a consequence whānau discussed their fear of answering questionnaires such as the SDQ, worrying what ‘they’ might be looking for, or that their children might be taken away from them. Their perceptions were compounded by some of the language used in the questionnaire, such as *assessment*, that was seen as objectionable and suspicious.

*“The intention will be well meaning. It will be, but the paradigm behind that intention, for me, always with European people, always comes from a deficit judgement of us as Māori people, because we never seem to quite make the grade according to their expectations or the standards that society has very heavily placed in, has in place in New Zealand in the 21st Century. They’re still judging us from a deficit mentality”.* (Māori participant ID4_3)

### Questions and more questions

Many questions were generated for the parents when reading through and reflecting on the questionnaire and as a consequence many found it a challenging process. For example, participants reported some questions were ambiguous and included words they would not use:

*“Nervous or clingy in new situation, easily loses confidence. I think these are two different things. They’re not the same. If he or she is clingy in a new situation, see somebody new, it’s not that they’re losing the confidence”.* (Indian participant ID1_7)

*“Often fights with other children or bullies them, is a bit ambiguous because people, especially nowadays, there’s so many different types of bullying, is it physical, is it verbal”.* (NZE participant ID83)

*“I think can be spiteful to others would be hard for a lot of people to understand, I don’t know I’d use the word spiteful for a four year old”.* (NZE participant ID78)

Participants also reported that the scoring system was not completely clear:

*“They’re saying, not true, or somewhat true, well what’s somewhat true? It’s either true or not true. If you mean somewhat true, well is my child nervous or clingy in these situations and easily loses confidence, how do you say somewhat true you know? What does that mean?”* (Māori participant ID77)

Furthermore, many participants reported they would prefer to use a translated version of the SDQ, with possible support from translators to complete it, and suggested inclusion of Māori words. This was not an issue raised by New Zealand European parents.

*“With me, I need someone to help me to complete the form. If possible, translate the form into Tongan version so I can complete it without any help”.* (Translated: Tongan participant ID23)

Some Māori participants suggested that by embedding more Māori words or terms the tool would be more acceptable and Māori would be more likely to engage with it. However, not all Māori agreed that this would be helpful, or even that a Māori translated tool would be the answer. Their responses stemmed from their stance that they did not support the overarching aim of a tick box exercise for screening children.

*“I think you can get all of these things from Māori whānau by just rephrasing it or it’s actually how you deliver it, not so much what you’re, if you want to ask, ‘Is your child considerate of other peoples’ feelings?’ You know are they, do you know, ‘Do they awhi awhi* < embrace > *their hoa* < friend > *?’ Most parents would say, ‘Yeah they do or sometimes.’”* (Māori participant ID77)

***“*** *If someone translates this, all it’s doing is just putting Māori words to a Pākehā <* New Zealander of European descent > *whakaaro* < way of thinking>*, and that is wrong. If you’re going to put something into Māori, make sure that there’s a tikanga* < custom > *Māori behind it, because this is not tikanga Māori”.* (Māori participant ID5_2)

Similarly, many participants, when asked, suggested that it would be helpful to complete the questionnaire with the nurse:

“*From a cultural perspective, for me being Tongan, addressing these straight up like that it’s not okay, eh, not, it’s not, you have to have a relationship, build relationship. And you have to be able to translate these questions into a similar content as in the Tongan, meaning the same but in a much more appropriate way. …. It’ll be a conversation. It’s not just a tick, tick, tick”.* (Tongan participant ID6_3)

### Timing of the questionnaire

This theme concerned the notion that children change quickly when they are aged between 4 and 5 and some considered the pre-school age to be too young for an assessment of their child’s strengths and difficulties. Similarly, some reported that more regular assessment would capture the changes that occur through the early school years.

*“She’s become more independent and she’s a bit more argumentative because she’s pushing those boundaries a bit more. So if I was to answer this now, I think there would be certainly some where she’d be scoring on a more frequent basis than she would have just over four when she was a bit more compliant”.* (NZE Participant ID83)

*“I guess for a lot of children it is a massive change* < going to school > *and that takes probably a good year to settle down before they get their heads round school. So I don’t know if there’s a programme of assessment throughout maybe sort of from four’s probably a good place to start but you probably don’t want to finish this until they’re probably seven or 10”.* (New immigrant participant, ID85)

### Multiple perspectives

Participants discussed the fact that different people, such as two parents, or parents and teachers, might provide different perspectives of the child. They recognised this could be due to children behaving differently depending on their environment. Some therefore felt that getting teachers’ perspectives would provide some balance to what the parents are reporting, although not all agreed with this approach and did not want teachers involved at all.

*“Some people look at different angle you know, child and some father look at different angle of child, mother looks a different angle you know and teachers they look. So I think more the people involved, the more reveal you know?”* (Asian parent, ID69)

*“And we have one teacher who’s obsessed with* < child name > *interpersonal skills, whereas the others are not nearly as concerned. So I’d be kind of saying I don’t want her doing it”.* (NZE participant ID11_5)

## Discussion

This study is the first to evaluate the cross-cultural acceptability and utility of the SDQ with a multi-ethnic sample of parents of pre-schoolers. Our findings showed people were unclear about the purpose and process of administration of the SDQ. They reported concerns that the timing and completion of a questionnaire did not permit the consideration of the children’s and their families/whānau context or cultural values and practices. In addition, contrary to widespread thinking that the SDQ is an easy and clear tool, our participants reported problems with words used in the questionnaire and found many questions confusing. Colleagues from Australia have also reported some questions to be confusing [11]. However as indicated in the introduction, that study is the only qualitative study that has explored the content validity of the SDQ – others have not specifically sought to examine this.

Validating outcome measures for use in different cultures/countries requires language translations and, if required, cultural adaptations [25]. The SDQ has been translated in over seventy different languages and there is a robust process in place to ensure the language equivalence of the tool, using forward – backward translation, as recommended by experts [29]. However, as discussed earlier, there has been only one paper that examined the cultural validity of the tool [11]. Consequently, across the world it seems that it is assumed that humans, by nature, are alike despite living in different cultures and, as such, a psychological theory (in this instance psychosocial attributes of children) developed in one culture has equal validity in another [25, 30]. Our study showed that parents from the Māori, Pacific Island, Asian, and new immigrant groups questioned the cultural validity of the SDQ. They commented that there was a lack of explicit consideration of children in their cultural context. Furthermore, many Māori parents advised that the process and tool appeared as a Pākehā (New Zealander of European descent) approach to labelling their children, and did not consider thinking about children in the context of their history. Thus, our study suggests that the SDQ has cross-cultural construct bias, in other words the construct measured is not identical or may have different meaning across cultural groups [31]. In addition, many parents from non-English backgrounds, as well as New Zealand European parents, commented that the words in the SDQ were not clear. Some would have liked access to a translated version of the SDQ or an interpreter. These findings were also reported by a study of New Zealand adolescents using mental health services and their families [32]. Their participants were concerned that the outcome measures used were developed elsewhere and may not fit the unique needs of different ethnic groups within New Zealand. Māori participants were also concerned that information arising from the measures might be politicised and misrepresented. Again, similar to our study, they showed that the cultural differences would impact on what may be considered as normal or acceptable.

The SDQ is supposed to be used as a self-completed questionnaire by parents and teachers. Used correctly, the SDQ should provide a very quick screen of children’s strengths and difficulties. This qualitative study showed that the process of the SDQ delivery is more complex with many parents commenting they were not supportive of a questionnaire approach and instead preferred a discussion with a nurse. Parents were also concerned that they were not able to qualify the context within which they were answering the questions. This is similar to findings of the use of the SDQ in Australia [11] as well as the aforementioned study of young people and their family in adolescent mental health services in New Zealand [32]. Stasiak and colleagues (2013) reported that questionnaires do not reflect the complexity of mental health or wellbeing, nor capture fluctuations in mental health. They found that a trusting relationship with the clinician administering the questionnaire should be established first through a face to face interaction. Others have similarly found that the process of care delivery is equally as important as outcome for Māori and Pasifika peoples [33]. These studies echo some of our participants’ suggestions in preferring a face to face approach to discuss their children’s strengths and difficulties.

Participants in our study discussed the notion that children change quickly as they develop over time and that ongoing monitoring of their strengths and difficulties would be helpful. The SDQ has different versions for children of older age groups and in many countries the tool is used longitudinally [34]. This enables an approach of surveillance of children, in which other domains (e.g. developmental delay, speech and language difficulties) and the wider context of the child are also considered [35]. The Before School Check in New Zealand includes these wider assessments of pre-school children [36], although currently longitudinal use of the SDQ is not in place.

Strengths of our study include the use of a multi-ethnic team of researchers both during data collection and analysis and the inclusion of a sizeable group of parents. However, the majority of our participants were female (85 %) and mothers (80 %). We were able to calculate the proportion of mothers that attended the Before School Check with their child in 2011 from the full de-identified national dataset, which was 81 %. Thus, our sample was representative of the population that completes the questionnaire routinely. Whilst the sample was not representative of fathers in New Zealand, there were a reasonable number who took part in the study (*n =* 10). In addition, differences between the views of mothers and fathers were not evident from the data. We were pleased with the number of participants from more rural areas. However, there were no participants from the middle or lower parts of the North Island or from the South Island of New Zealand. Findings are therefore not transferable to these groups. The quantitative psychometric properties were examined in two separate studies (papers in preparation) [37]), which utilised SDQ data from the 2011 New Zealand national Before School Check database. This included factor and Rasch analyses to examine the structural validity, test and item bias; calculations of normative values for the New Zealand pre-school population; as well as concurrent validity examined with 225 Māori children. Due to space we cannot report these studies here.

## Conclusion

This study posed many challenges to the cross-cultural acceptability and utility of the SDQ in the New Zealand context. Parents struggled with understanding the tool’s purpose and had preference for face to face interactions with clinicians. We recommend further work is undertaken at a clinical level to explain the value of the tool and alleviate parents’ concerns, and to offer face to face support when completing the SDQ. Similarly, it is important that those for whom English is not their first language are offered the appropriate language version of the SDQ and, when required, support from interpreters. Furthermore, work is required to develop a tool that is culturally appropriate and valid.

## Abbreviations

SDQ: Strengths and Difficulties Questionnaire
